# Necroptosis in asthma: a critical driver of immune dysregulation and airway remodeling

**DOI:** 10.3389/fimmu.2026.1783960

**Published:** 2026-06-23

**Authors:** Feng-Xian Ni, Shao-Cong Ren, Jie Hu, Pei-Sheng Chen, Hui-Hui Chen, Dong-Hui Huang, Ze-Bo Jiang

**Affiliations:** 1 Zhuhai Hospital of Integrated Traditional Chinese & Western Medicine, Zhuhai, Guangdong, China; 2 Zhuhai Hospital Affiliated to Faculty of Chinese Medicine, Macau University of Science and Technology, Zhuhai, Guangdong, China

**Keywords:** airway remodeling, asthma, DAMPs, necroptosis, PANoptosis, RIPK3, steroid resistance

## Abstract

Asthma is a heterogeneous chronic airway disease traditionally framed within the context of dysregulated adaptive T helper 2 (Th2) immunity. However, this paradigm insufficiently explains disease chronicity, structural remodeling, and the limited efficacy of corticosteroids in specific endotypes. The discovery of necroptosis, a regulated form of lytic cell death governed by receptor-interacting protein kinase 1 and 3 (RIPK1/RIPK3) and mixed-lineage kinase domain-like protein (MLKL), introduces a transformative perspective. This review posits that necroptosis is not a passive endpoint but an active pathogenic hub in asthma. We systematically dissect how asthma-relevant triggers (allergens, viruses, pollutants) activate necroptosis in a cell-specific manner. We detail the consequences: damage-associated molecular pattern (DAMP)-driven initiation and amplification of type 2 inflammation via epithelial death, the establishment of a neutrophilic, steroid-refractory milieu through immune cell necroptosis, and direct contributions to airway remodeling. Furthermore, we discuss the interconnectedness of necroptosis with pyroptosis and apoptosis within the PANoptosis framework, and acknowledge the limitations of current evidence derived largely from preclinical models. Finally, we explore therapeutic implications, advocating for patient stratification using necroptosis biomarkers and evaluating novel pharmacological inhibitors. By integrating necroptosis into the asthma pathophysiological network, this review provides a unified mechanistic framework that bridges initial insult to sustained inflammation and tissue remodeling, unveiling novel avenues for disease-modifying therapies.

## 1 Introduction

Asthma remains a global health burden, characterized by variable airflow obstruction, bronchial hyperresponsiveness, and chronic inflammation (1). The dominant pathophysiological model has long centered on aberrant adaptive immunity, particularly the dysregulation of type 2 helper T (Th2) cells and their signature cytokines—interleukin (IL)-4, IL-5, and IL-13 (2). This “Th2-high” paradigm successfully explains allergic eosinophilic inflammation and underpins the mechanism of targeted biologics such as anti-IgE (omalizumab) and anti-IL-4Rα/IL-13 (dupilumab) (3–5). Nevertheless, a significant proportion of patients, particularly those with severe, steroid-resistant, or non-eosinophilic (neutrophilic) asthma, derive suboptimal benefit from these strategies (6–8). This clinical gap underscores a fundamental limitation: our canonical models fail to fully encapsulate the drivers of disease persistence, heterogeneity, and the irreversible structural changes termed airway remodeling.

The critical missing link may reside not in how immune cells live and function, but in how they die, and how structural cells succumb to injury. Apoptosis, a programmed, immunologically silent cell death, has been studied in asthma but does not account for the intense, self-perpetuating inflammation observed (9, 10). In contrast, necroptosis is a genetically encoded, caspase-independent form of regulated necrosis that is potently pro-inflammatory (11, 12). Its execution involves a defined molecular cascade: upon specific signaling (e.g., from death receptors under caspase-8 inhibition), RIPK1 and RIPK3 form a functional amyloid signaling complex termed the necrosome (13). RIPK3 then phosphorylates the terminal effector MLKL, triggering its oligomerization, membrane translocation, and pore formation, leading to cytolysis and the release of intracellular contents (14).

Critically, necroptosis is a highly immunogenic process (11). Lytic rupture releases a plethora of intracellular components known as damage-associated molecular patterns (DAMPs), including high-mobility group box 1 (HMGB1), ATP, mitochondrial DNA, and S100 proteins (15–17). These molecules act as potent endogenous adjuvants, activating pattern recognition receptors (e.g., Toll-like receptors, TLRs; the NLRP3 inflammasome) on innate immune sentinels, thereby fueling further cytokine production and leukocyte recruitment (18–20).

The asthmatic airway presents a unique microenvironment conducive to necroptosis (21–23). It is a site of recurrent exposure to exogenous triggers (allergen proteases, viruses, pollutants) capable of initiating death receptor signaling or inhibiting caspase-8. It also features profound oxidative stress and cytokine milieus (e.g., TNF-α, IFNs) that can prime the necroptotic machinery. Furthermore, key cellular actors in asthma—bronchial epithelial cells, alveolar macrophages, and eosinophils—express core components of the necroptotic pathway.

It is important to recognize that necroptosis does not operate in isolation. The asthmatic airway is a site of complex interplay among multiple regulated cell death (RCD) pathways. Pyroptosis, mediated by gasdermin family proteins and often downstream of NLRP3 inflammasome activation, has been implicated in epithelial barrier dysfunction and neutrophilic inflammation (24–26). Ferroptosis, an iron-dependent lipid peroxidation-driven death, is emerging as a contributor to airway epithelial injury under conditions of oxidative stress and glutathione depletion (27, 28). Autophagy can play dual roles, either promoting cell survival or facilitating other death programs (29). Recent conceptual advances have framed these intersecting pathways under the term PANoptosis, which recognizes that RCD molecules (e.g., caspases, RIPKs, gasdermins) can form higher-order complexes that execute a hybrid form of cell death with mixed features (30, 31). Throughout this review, while we focus on necroptosis as a critical driver, we emphasize its integration into this broader RCD network.

Most mechanistic insights into necroptosis in asthma derive from *in vitro* cell culture systems and murine models. While these have been invaluable, species-specific differences exist in the regulation of key effectors such as MLKL and RIPK3, and the inflammatory microenvironment of the human asthmatic lung may differ substantially from that of challenged mice (32). Moreover, obtaining longitudinal human airway tissue for dynamic assessment of necroptotic activity remains a significant technical challenge. Wherever possible, we note the translational gap between preclinical findings and human disease, and highlight emerging studies using human bronchial biopsies, sputum cells, and ex vivo precision-cut lung slices.

This review advances the hypothesis that necroptosis serves as a critical mechanistic linchpin in asthma, directly translating diverse environmental and immunological insults into sustained immune dysregulation and tissue remodeling. We will systematically explore the molecular triggers of necroptosis within the asthmatic lung, delineate its cell-type-specific consequences, and elucidate how it orchestrates a pathogenic immune microenvironment that perpetuates inflammation, drives clinical heterogeneity, and undermines therapeutic response. Finally, we will critically appraise the translational potential of targeting this pathway, proposing a framework for biomarker-driven patient stratification and novel intervention strategies aimed at achieving disease modification in asthma.

## 2 Molecular mechanisms of necroptosis: unique activation within the asthmatic milieu

**Figure 1:**
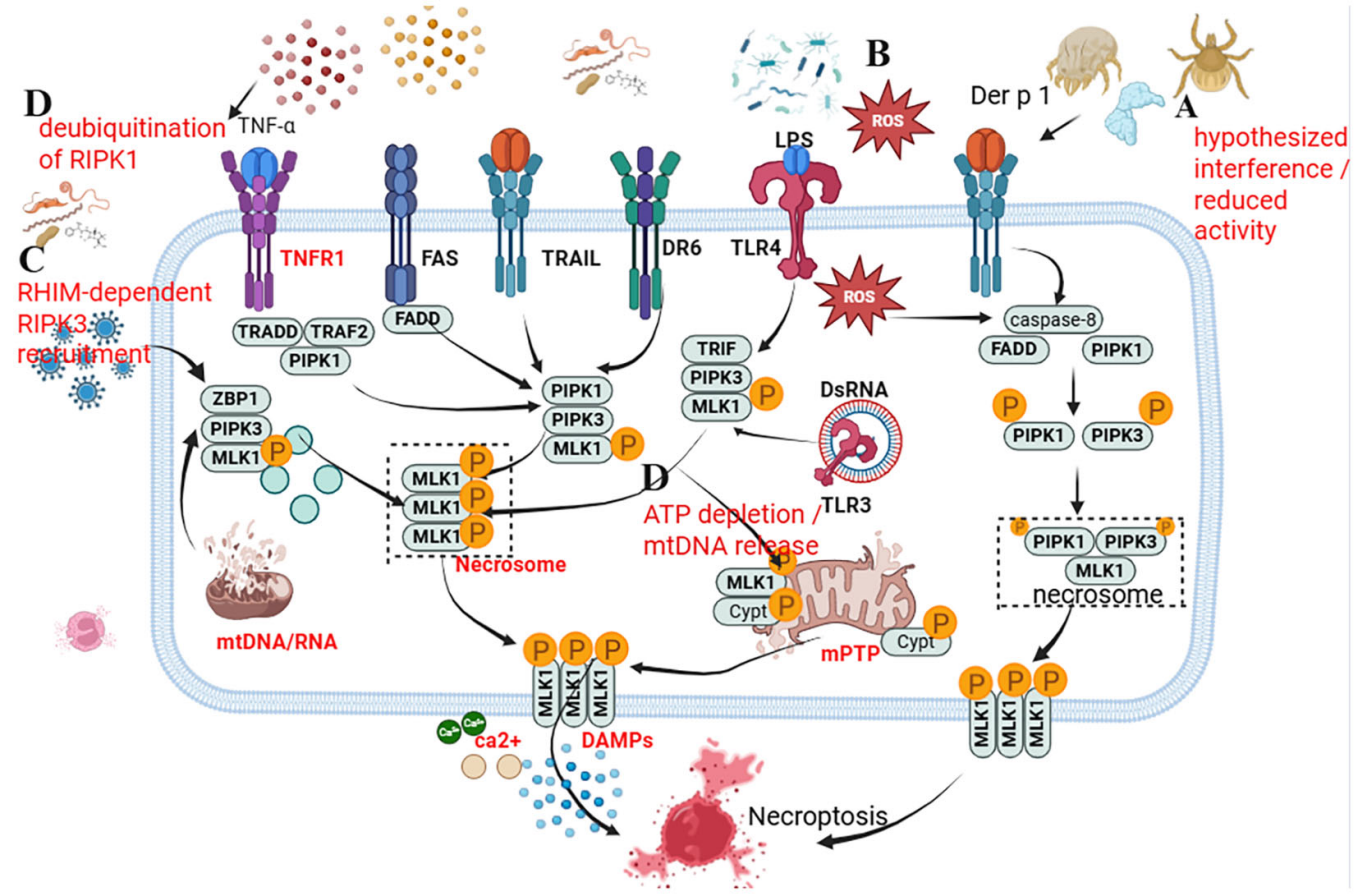
Molecular mechanisms of necroptosis and its unique activation within the asthmatic milieu. The canonical necroptosis pathway is initiated by death receptor (e.g., TNFR1) ligation. Under homeostatic conditions, caspase-8 activity promotes apoptosis and suppresses necroptosis. In the asthmatic airway, multiple triggers converge to dismantle these safeguards and hyperactivate the pathway. **(A)** Allergen−derived proteases (e.g., Der p 1) are hypothesized to interfere with the apoptotic machinery, reducing caspase−8 activity and lowering the threshold for necroptosis. **(B)** Oxidative stress (ROS/RNS) post−translationally modifies caspase−8 (e.g., S−nitrosylation, tyrosine nitration) and depletes glutathione, further compromising caspase−8 function. **(C)** Viral infections engage TLR3 and ZBP1 via RHIM−domain interactions, allowing TRIF or ZBP1 to directly recruit and activate RIPK3 independently of RIPK1. **(D)** Inflammatory cytokines (TNF−α, IFNs) and oxidative stress upregulate deubiquitinases (e.g., CYLD) and downregulate cIAP1/2, shifting RIPK1 ubiquitination from pro−survival (K63−/M1−linked) to a pro−death state. **(E)** Metabolic stress and mitochondrial dysfunction reduce ATP availability (impairing apoptosis) and release mitochondrial DAMPs. The convergence of these signals leads to necrosome formation (RIPK1−RIPK3 complex), phosphorylation of MLKL, and its oligomerization. MLKL pores disrupt the plasma membrane, causing lytic release of DAMPs (HMGB1, ATP, mtDNA) and alarmins (TSLP, IL−33, IL−25), which fuel inflammation.

The pathogenesis of asthma has undergone a paradigm shift with the recognition of RCD as a central driver of inflammation, moving beyond the traditional focus solely on immune cell activation (33). Among these pathways, necroptosis, alongside pyroptosis, is distinguished by its intensely pro−inflammatory nature, as both lead to the lytic release of intracellular contents (15). Its core machinery, comprising receptor-interacting protein kinase 1 (RIPK1), RIPK3, and mixed-lineage kinase domain-like protein (MLKL), is not a blunt instrument but an elegantly regulated signaling cascade with built-in checkpoints (34). A central thesis of this review is that the asthmatic lung microenvironment does not merely trigger necroptosis; it systematically dismantles its regulatory safeguards and hyper-activates its triggers, creating a pathological landscape where necroptosis becomes a predominant mode of cell death. This section details the molecular intricacies of the necroptotic pathway and the multifarious ways in which asthma-specific factors subvert this system to fuel disease (**Figure 1**).

### 2.1 The canonical RIPK1-RIPK3-MLKL axis: a precariously balanced signaling cascade

The most well-characterized pathway to necroptosis is initiated by members of the death receptor superfamily, such as Tumor Necrosis Factor Receptor 1 (TNFR1), Fas (CD95), and TRAIL receptors (35–37). Under homeostatic conditions, ligation of TNFR1 by TNF-α leads to the rapid assembly of a membrane-associated multiprotein complex, known as Complex I. This complex includes TRADD, TRAF2/5, cellular inhibitor of apoptosis proteins 1 and 2 (cIAP1/2), and RIPK1. Here, RIPK1 undergoes extensive K63-linked and linear (M1-linked) polyubiquitination, primarily mediated by cIAP1/2 and the linear ubiquitin chain assembly complex (LUBAC), respectively (38, 39). This ubiquitin coating serves as a scaffold for recruiting downstream kinases like TAK1 and IKK complexes, ultimately activating the NF-κB and MAPK pathways to promote inflammatory gene transcription and cell survival (40). In this context, RIPK1 functions as a scaffold, and its kinase activity is suppressed.

The switch from pro-survival to pro-death signaling hinges on the integrity of Complex I. When TNFR1 signaling is robust, cell survival prevails. However, when Complex I signaling is disrupted—due to insufficient ubiquitination, increased deubiquitinase activity (e.g., CYLD), or downregulation of cIAP1/2—RIPK1 is released from Complex I into the cytosol, where it assembles pro-death complexes. These cytosolic complexes can take distinct forms. When caspase-8 is active, it is recruited together with FADD to form Complex IIa (or Complex IIb, depending on the presence of RIPK1), leading to apoptosis (41, 42). Within this setting, caspase-8 cleaves and inactivates RIPK1 and RIPK3, thereby suppressing necroptosis (43). Thus, caspase-8 serves as the primary molecular brake on necroptosis.

When caspase-8 activity is absent, pharmacologically inhibited, or overwhelmed, the cytosolic complex morphs into the necrosome (sometimes designated Complex IIc) (44, 45). In this configuration, RIPK1 transitions from a scaffold to an active kinase. Importantly, while RIPK1 does not directly phosphorylate RIPK3, it is essential for the stabilization and full activation of RIPK3 in most contexts (46, 47). Through their RIP homotypic interaction motifs (RHIMs), RIPK1 and RIPK3 engage in a reciprocal activation process. RIPK3 can phosphorylate RIPK1, and the kinase activity of RIPK1 facilitates the phosphorylation of RIPK3 at critical residues (e.g., Ser227 in humans) (48, 49). This process often leads to the formation of amyloid-like fibrils that serve as a potent signaling platform for signal amplification.

The committed execution step is RIPK3-mediated phosphorylation of MLKL at specific residues (e.g., Thr357 and Ser358 in humans) (50). This triggers a conformational change in MLKL, exposing its N-terminal four-helical bundle (4HB) domain. Phosphorylated MLKL oligomerizes and translocates to the plasma membrane, where it inserts to form pores or activates ion channels (e.g., TRPM7). The resultant loss of ionic homeostasis leads to cellular swelling (oncosis) and plasma membrane rupture, releasing a flood of DAMPs (51).

### 2.2 Asthma-specific modulators of the necroptotic switch: engineering a perfect storm

The asthmatic lung is not a passive tissue but a dynamically hostile microenvironment that actively reprograms cellular fate decisions (52). It achieves this by simultaneously applying multiple pressures on the precise regulatory nodes of the necroptosis pathway, effectively “arming” the machinery and “disabling” the safety catches.

#### 2.2.1 Strategic disabling of the brake: caspase-8 inhibition

The inhibition of caspase-8 is the most critical permissive signal for necroptosis (53). Asthma pathophysiology provides an arsenal of tools to achieve this.

##### 2.2.1.1 Allergen-derived proteases as molecular saboteurs

Major allergens such as the house dust mite cysteine protease Der p 1 possess intrinsic protease activity capable of disrupting epithelial tight junctions (54). Beyond barrier disruption, it has been hypothesized that certain allergen proteases may interfere with the apoptotic machinery, including potential effects on caspase-8 or its adaptor FADD, thereby lowering the threshold for necroptosis (55). However, direct biochemical evidence for cleavage of these molecules by Der p 1 remains limited, and the field relies heavily on functional inference from studies showing reduced apoptotic capacity and increased necroptotic markers following allergen exposure. Similarly, proteases from *Alternaria alternata*, a potent trigger for severe asthma, can induce epithelial cell death with necrotic morphology (56). These observations collectively suggest that allergen proteases create a permissive environment for necroptosis, though the precise molecular targets await further validation.

##### 2.2.1.2 Oxidative stress

A hallmark of asthma, oxidative stress generates a flood of reactive oxygen and nitrogen species (ROS/RNS) (57, 58). These molecules can modify death signaling pathways at multiple levels. For example, S−nitrosylation of caspases, including caspase−3, has been well documented, and it is plausible that caspase−8 is similarly modified under the high−NO conditions of the asthmatic airway, although direct evidence for caspase−8 S−nitrosylation in asthma is lacking (59). Peroxynitrite (ONOO^-^), formed from superoxide and NO, can cause tyrosine nitration of various proteins, potentially altering the function of death−signaling molecules (60). Furthermore, oxidative stress depletes cellular glutathione (GSH) pools, altering the redox state and affecting the activity of redox−sensitive enzymes involved in death signaling (61). Collectively, the asthmatic airway creates a biochemical environment in which caspase−8 activity may be compromised through multiple mechanisms: direct S-nitrosylation (plausible but not yet directly demonstrated in asthma), tyrosine nitration by peroxynitrite (62, 63), and depletion of glutathione pools (64). These modifications collectively lower the necroptosis threshold (65). The asthmatic airway, particularly during exacerbations, is rich in these modifications, creating a milieu where the caspase-8 brake is chemically compromised.

##### 2.2.1.3 Viral commandering of cell death pathways

Rhinovirus (RV) and respiratory syncytial virus (RSV), common triggers of acute asthma exacerbations, have evolved sophisticated mechanisms to inhibit host cell apoptosis to facilitate viral replication (66). Certain RV serotypes and RSV proteins can directly cleave or bind to caspase-8 activators like RIPK1 or FADD (67). This viral strategy to delay apoptosis inadvertently removes the blockade on necroptosis. Consequently, the infected cell, once it has served its purpose or is detected by intracellular sensors, may be shunted towards a highly inflammatory necroptotic death, releasing virions and DAMPs simultaneously to amplify inflammation and worsen airway symptoms.

#### 2.2.2 Rewriting the ubiquitin code: shifting RIPK1 from scaffold to executioner

The fate of RIPK1 is dictated by a dynamic “ubiquitin code”. Inflammatory cytokines (TNF-α, IL-1β) and oxidative stress in asthma can upregulate the deubiquitinase CYLD, which removes K63-linked ubiquitin chains from RIPK1, facilitating its release from Complex I and incorporation into the necrosome (68, 69). This deubiquitination is a decisive step that liberates RIPK1 from Complex I, allowing its translocation to the cytosol and facilitating its incorporation into the necrosome. Concurrently, asthma-related inflammation can lead to the downregulation or inhibition of cIAP1/2, the E3 ligases responsible for the initial protective ubiquitination (70). This dual hit—enhanced deubiquitination and impaired ubiquitination—powerfully biases RIPK1 toward its pro-death, kinase-active conformation.

The LUBAC complex, which adds linear M1-linked ubiquitin chains, is generally considered protective in TNF signaling by stabilizing Complex I. However, dysregulation of LUBAC components (HOIP, HOIL-1, SHARPIN) has been linked to inflammatory pathologies in other diseases (53). However, whether LUBAC integrity is perturbed in the asthmatic airway, thereby contributing to unstable complex formation and facilitating the transition to necroptosis, remains an open question that warrants further investigation.

#### 2.2.3 Metabolic stress: fueling the wrong kind of fire

The inflamed asthmatic airway is a site of metabolic competition and reprogramming, which indirectly promotes necroptosis. Activated immune cells switch to aerobic glycolysis (Warburg effect), consuming local glucose and creating zones of nutrient deprivation for structural cells. Apoptosis is an energy (ATP)-dependent process. Under metabolic stress, ATP depletion can lead to “apoptotic failure, ” allowing ATP-independent necroptosis to become the default death pathway (71). Furthermore, mitochondrial dysfunction in asthma releases DAMPs (e.g., mtDNA) that can amplify inflammatory and necroptotic signaling (15, 72–74).

#### 2.2.4 Bypassing the traditional checkpoint: direct engagement of alternative triggers

Asthma exacerbations often involve triggers that can activate necroptosis without the classical TNF/death receptor cascade, making the pathway responsive to a wider range of insults.

##### 2.2.4.1 Toll-like receptor 3 engagement

Viral double-stranded RNA (dsRNA), a ubiquitous product of viral replication during RV exacerbations, is a potent ligand for TLR3. The TLR3 adaptor protein, TRIF, contains a RHIM domain. Upon dsRNA binding, TRIF can directly recruit and activate RIPK3 via RHIM-RHIM interaction, forming a necrosome independent of RIPK1. This provides a direct mechanistic line from viral infection to necroptotic cell death in epithelial cells and macrophages, driving antiviral inflammation that can become pathological in asthma. Studies using TRIF-deficient mice or RHIM-mutant TRIF constructs have validated the importance of this axis in lung inflammatory responses (75).

##### 2.2.4.2 Z-DNA binding protein 1 (ZBP1) as an intracellular sentinel

ZBP1 (also known as DAI) is a cytosolic nucleic acid sensor that recognizes Z-form nucleic acids, which can be produced during viral replication or cellular stress. Critically, ZBP1 also contains a RHIM domain (76). Sensing of viral RNA or endogenous Z-RNA (e.g., from retrotransposons activated during inflammation) can lead to ZBP1 oligomerization and direct recruitment of RIPK3, forming a RIPK1-independent necrosome (77). This pathway is increasingly recognized as a key mediator of inflammatory cell death in response to viral infection and may be particularly relevant in severe, infection-triggered asthma. Notably, recent work has shown that in airway macrophages, the PTRF–IL-33–ZBP1 axis mediates house dust mite-induced necroptosis, linking allergen exposure to this alternative pathway (78). Furthermore, metabolic changes can regulate ZBP1 expression, creating a potential link between the metabolic stress of asthma and susceptibility to this form of necroptosis (79).

##### 2.2.4.3 Interferon priming

Type I and II interferons (IFNs), commonly upregulated during viral asthma exacerbations, can dramatically upregulate the expression of key necroptosis components, including RIPK3, MLKL, and ZBP1 (80–82). This “priming” effect lowers the activation threshold for necroptosis, making cells exquisitely sensitive to subsequent triggers. An IFN-rich environment, therefore, sets the stage for rampant necroptosis upon secondary insult.

##### 2.2.4.4 The amplification loop: DAMPs beget more necroptosis

DAMPs released from necroptotic cells (HMGB1, ATP, S100 proteins, DNA) activate neighboring cells via PRRs like TLR4 and P2X7, inducing production of more TNF-α and other cytokines, providing Signal 2 for NLRP3 inflammasome activation (leading to IL-1β release), and further upregulate necroptosis pathway components (20). This creates a self-sustaining wave of inflammation and cell death through airway tissue. The asthmatic lung is a master manipulator of cell death pathways. It employs a multi-pronged strategy involving proteolytic disarmament, oxidative modification, metabolic competition, and alternative pathway activation to co-opt the powerful necroptotic machinery. This transforms a potentially protective host defense mechanism into a relentless engine of tissue damage and chronic inflammation. The subsequent chapters will explore the consequences of this hijacked machinery in specific lung cell populations.

### 2.3 The broader landscape of regulated cell death: necroptosis in the PANoptosis paradigm

The boundaries between necroptosis and other RCD pathways are increasingly recognized as fluid. For instance, caspase-8, the critical brake on necroptosis, can also cleave gasdermin D to induce pyroptosis-like membrane rupture under certain conditions (26, 83–86). Conversely, the MLKL pores formed during necroptosis can activate the NLRP3 inflammasome, bridging necroptosis to pyroptotic IL-1β release (87). These observations have led to the concept of PANoptosis, in which molecular components from multiple RCD pathways (caspases, RIPKs, gasdermins) assemble into higher−order complexes (PANoptosomes) that execute a hybrid form of inflammatory cell death (30). Notably, studies in evolutionarily ancient organisms such as *Hydra* have demonstrated that pyroptotic signaling modules are mechanistically versatile and can be engaged through caspase-dependent pathways, suggesting that the fluid boundaries between cell death programs are deeply conserved (88). In the asthmatic airway, it is plausible that such complexes form in response to complex triggers (e.g., viral infection combined with oxidative stress). For the purpose of this review, we focus on necroptosis as a key experimentally tractable driver, while acknowledging that the true pathological landscape likely involves cross−talk among multiple RCD programs.

## 3 Cell-type-specific roles in asthma: the actors and their demise

**Figure 2:**
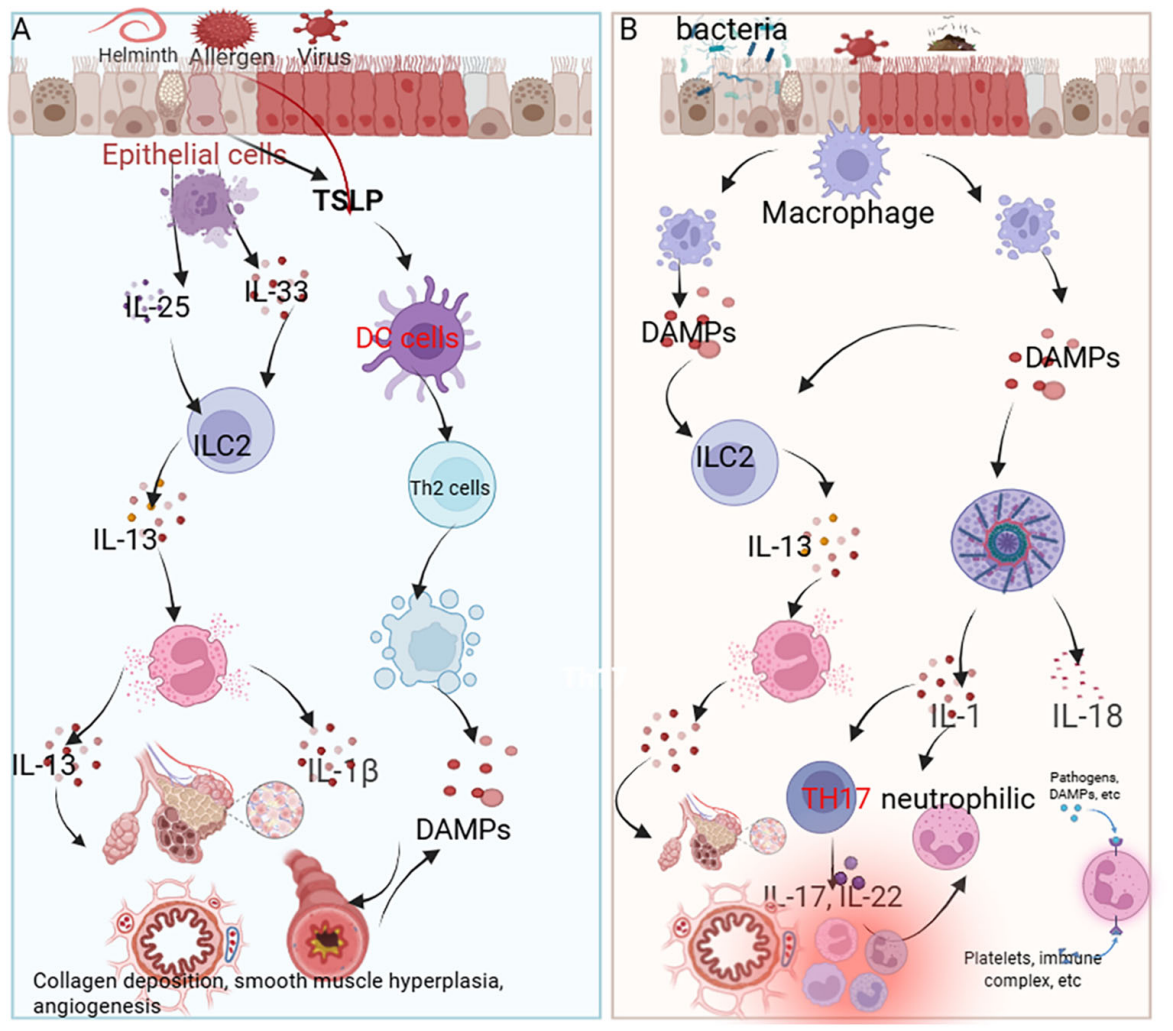
Necroptosis orchestrates distinct immune microenvironments driving asthma endotypes and airway remodeling. Divergent patterns of necroptotic activity underlie the pathogenesis of major asthma endotypes, with bidirectional feedback loops perpetuating disease. **(A)** Type 2-High (Eosinophilic) endotype: Allergen−triggered epithelial necroptosis releases alarmins (TSLP, IL−33, IL−25), activating ILC2s and dendritic cells to polarize Th2 cells. Th2 cytokines (IL−4, IL−5, IL−13) drive IgE production, eosinophil recruitment, and mucus hypersecretion. Recruited eosinophils can undergo secondary necroptosis, releasing cytotoxic granules that further damage the epithelium, creating an eosinophil−epithelial injury loop. **(B)** Neutrophilic, steroid−resistant endotype: Macrophage necroptosis triggered by viruses, pollutants, or bacteria releases DAMPs (ATP, mtDNA, HMGB1), which activate the NLRP3 inflammasome and produce IL−1β and IL−18. IL−1β drives neutrophil chemoattractants and Th17 differentiation; IL−17 amplifies neutrophilic inflammation. This DAMP−inflammasome axis is associated with corticosteroid resistance, and neutrophil necroptosis further fuels the cycle. Chronic necroptotic activity from both endotypes generates a pro−fibrotic milieu, promoting subepithelial fibrosis, airway smooth muscle hyperplasia, angiogenesis, and neural plasticity, leading to fixed airway obstruction.

The pathophysiology of asthma is not a monologue but a complex drama involving a diverse cast of cellular actors. Necroptosis, far from being a nonspecific destructive event, is a discerning director that eliminates specific characters, each with a unique role, thereby irrevocably altering the plot of the immune response. The impact of necroptosis is exquisitely defined by the inherent susceptibility of different cell types—shaped by their expression of receptors, sensors, and metabolic pathways—and, most importantly, by the distinct immunogenic signature released upon their lytic demise. This cell-type-specific orchestration of death is what translates a molecular pathway into the heterogeneous clinical phenotypes of asthma (**Figure 2**). This chapter will dissect the necroptotic fate of the principal cellular players in the asthmatic lung, detailing the triggers, molecular execution, and the catastrophic downstream consequences that collectively drive immune dysregulation and tissue remodeling.

### 3.1 The sentinel breach: bronchial epithelial cell necroptosis

The pseudostratified columnar epithelium lining the airways is far more than a passive barrier; it is a dynamic sensory and secretory organ, integral to immune homeostasis. Its strategic position makes it the primary target for inhaled insults, and its necroptosis represents a pivotal sentinel event that can initiate and amplify the asthmatic cascade through multiple, interconnected mechanisms.

#### 3.1.1 Triggers and molecular execution in the epithelium

The epithelial cell is continuously exposed to a hostile environment. As detailed in Chapter 2, several asthma-relevant triggers converge on the epithelial cell to promote necroptosis. Allergen-derived proteases, such as Der p 1 from house dust mite and proteases from Alternaria alternata, can disrupt epithelial barrier integrity and have been functionally associated with reduced apoptotic capacity, thereby creating a permissive state for necroptosis (89). Although direct biochemical evidence for Der p 1-mediated cleavage of caspase-8 or FADD in human airway epithelium remains limited, multiple studies report that allergen exposure leads to increased expression of RIPK3 and MLKL and decreased caspase-8 activity, suggestive of a shift toward necroptotic cell death (89, 90).

Viral infections, particularly with rhinovirus (RV) and respiratory syncytial virus (RSV), provide an even more direct trigger. Viral double−stranded RNA engages TLR3, whose adaptor TRIF contains a RHIM domain capable of recruiting RIPK3 independently of RIPK1 (66). This pathway has been documented in primary human bronchial epithelial cells infected with RV, leading to RIPK3−dependent, MLKL−mediated cell death and release of viral particles (91). Additionally, intracellular viral sensors such as RIG−I and MDA5 can upregulate ZBP1 expression, priming the epithelium for ZBP1−dependent necroptosis (92, 93).

The asthmatic epithelium also operates under chronic oxidative stress. Reactive oxygen and nitrogen species (ROS/RNS) generated from inflammatory cells and environmental pollutants deplete intracellular glutathione and can post−translationally modify key death regulators, including caspase−8 (94). While direct evidence for caspase−8 nitrosylation in asthmatic epithelium is lacking, functional studies demonstrate that exogenous oxidative stress reduces caspase−8 activity and sensitizes airway epithelial cells to necroptotic stimuli *in vitro* (89, 95). Collectively, these diverse triggers—allergen proteases, viral sensors, and oxidative stress—converge to dismantle the apoptotic brake, allowing the formation of the necrosome and execution of MLKL−dependent membrane rupture.

#### 3.1.2 Consequences: a multifaceted catastrophe

Necroptosis of bronchial epithelial cells unleashes a cascade of pathological consequences. First, the lytic loss of epithelial cells directly compromises barrier integrity. Tight junction proteins (ZO−1, occludin) and adherens junction proteins (E−cadherin) are disrupted, leading to increased paracellular permeability—a state often termed “leaky airway” (96). This barrier defect facilitates deeper penetration of aeroallergens, pathogens, and pollutants into the subepithelial space, where they encounter antigen-presenting cells and sensory nerves (18). Plasma exudation introduces serum proteins such as fibrinogen and albumin into the airway lumen, where under oxidative conditions they may form neoantigens, potentially driving autoimmune responses in severe asthma (97–99).

Second, necroptotic epithelial cells release a pre-formed “triad of alarmins”—TSLP, IL−25, and IL−33—in a non-conventional, instantaneous manner. TSLP programs dendritic cells to promote a Th2 differentiation and directly activates mast cells and group 2 innate lymphoid cells (ILC2s) (100). IL−33, a nuclear cytokine, signals through the ST2 receptor on ILC2s, Th2 cells, mast cells, and eosinophils, driving prolific production of IL-5 and IL-13 (101). IL−25 similarly expands ILC2 populations and amplifies type 2 cytokine output (102). The coordinated release of these alarmins from necroptotic epithelium establishes a potent innate type 2 inflammatory axis within hours, independent of adaptive immunity. This mechanism may explain the rapid onset of symptoms upon allergen challenge and the presence of type 2 inflammation in young children prior to full immune maturation.

Third, epithelial necroptosis bridges to neutrophilic inflammation through DAMP-mediated inflammasome activation. The lytic release of ATP acts on P2X7 receptors on neighboring cells, triggering potassium efflux—a key signal for NLRP3 inflammasome assembly (103). Concurrently, oxidized mitochondrial DNA (mtDNA) released from damaged mitochondria can directly bind and activate NLRP3 (104). Active NLRP3 inflammasome drives caspase-1-mediated processing of pro-IL-1β and pro-IL-18 into their mature forms. IL-1β then induces neutrophil chemoattractants (e.g., CXCL8) and promotes Th17 differentiation, while IL-18 synergizes with IL-12 to promote IFN-γ production (105). Thus, a single event of epithelial necroptosis possesses the dual capacity to initiate both type 2 (via alarmins) and type 1/neutrophilic (via inflammasome) responses, potentially explaining the mixed granulocytic inflammation frequently observed in severe asthma.

### 3.2 Macrophages: from regulators to incendiaries

Alveolar macrophages (AMs) and interstitial macrophages are the professional phagocytes of the lung, tasked with maintaining sterility, resolving inflammation, and promoting repair (106). Their necroptosis represents a catastrophic failure of these regulatory duties, transforming them from peacekeepers into potent instigators of persistent inflammation (107).

#### 3.2.1 Triggers and susceptibility

Macrophages exposed to the full repertoire of necroptotic machinery and are constantly exposed to DAMPs released from necroptotic epithelial cells (ATP, HMGB1, DNA) (108, 109). These DAMPs can prime macrophages via TLR4 (for HMGB1) and provide activation signals via P2X7 (for ATP) (110). During viral exacerbations, macrophages can be directly infected; intracellular viral RNA activates the ZBP1-RIPK3 axis, leading to RIPK-dependent necroptosis (111). Macrophages are also major producers of TNF-α. In an environment where caspase-8 is inhibited by oxidative stress or viral proteins, autocrine or paracrine TNF-α signaling can tip the balance towards auto-necroptosis (112). Interferon-γ (IFN-γ), often elevated in severe asthma, is a powerful primer that upregulates RIPK3 and MLKL expression, dramatically lowering the activation threshold for necroptosis (113–115).

#### 3.2.2 Consequences: the loss of control

Necroptosis of macrophages leads to a critical failure in inflammatory resolution. Under homeostatic conditions, macrophages efficiently clear apoptotic cells via efferocytosis, an anti-inflammatory process that promotes production of TGF-β, IL-10, and pro-resolving lipids (116). A macrophage undergoing necroptosis is incapable of this clearance. Consequently, apoptotic cells (e.g., from eosinophils or neutrophils) remain uncleared and progress to secondary necrosis, releasing their own cytotoxic and pro−inflammatory contents. This establishes a self−amplifying vicious cycle: impaired clearance leads to more necrotic debris, which releases additional DAMPs that further trigger macrophage death and perpetuate inflammation—a key proposed mechanism for chronic, non−resolving inflammation in asthma (117).

Simultaneously, necroptotic lysis of macrophages unleashes a potent burst of pre-formed inflammatory mediators. As cellular “cytokine factories, ” macrophages store large quantities of pro−IL−1α, pro−IL−1β, and TNF−α, which are released in an uncontrolled manner upon lytic death. IL−1α itself acts as a DAMP by signaling through the IL-1 receptor to drive further inflammation (118). The release of high−mobility group box 1 (HMGB1) is particularly consequential; in its oxidized form, HMGB1 sustains inflammation by engaging receptors such as RAGE and TLR4 (118–120). Together, these events create a persistently inflammatory, DAMP−saturated microenvironment that relentlessly activates neighboring structural and immune cells, thereby driving and sustaining the neutrophilic and steroid−resistant aspects of severe asthma.

In the healthy lung, a functional equilibrium exists between pro-inflammatory (M1-like) and pro-resolving/repair-oriented (M2-like) macrophage populations. Necroptosis disrupts this balance by selectively depleting the macrophage compartment. Furthermore, the ensuing storm of DAMPs pushes any surviving macrophages toward a sustained pro-inflammatory M1 phenotype (121). M2 macrophages are critical for tissue repair, as they produce essential growth factors such as VEGF and PDGF and contribute to inflammation resolution through IL-10 secretion and active efferocytosis. Their loss or functional impairment directly contributes to impaired wound healing and the unchecked progression of inflammatory damage into structural remodeling (122–124).

### 3.3 Eosinophils: a toxic and explosive demise

Eosinophils are the signature effector cells of allergic asthma, armed with granules containing cationic proteins. Their conventional, apoptotic death followed by macrophage efferocytosis is a silent process designed to limit tissue damage. Necroptosis subverts this safe disposal system with explosive consequences (125).

#### 3.3.1 Triggers for eosinophilic necroptosis

Eosinophils express death receptors (e.g., TNFR1, Fas) can be sensitive to TNF-α. More intriguingly, the very signals that activate and prolong eosinophil survival may prime them for necroptosis (126). IL-5 and GM-CSF, key eosinophilopoietins, activate the PI3K/Akt and JAK/STAT pathways, which can cross-talk with cell survival/death machinery (17, 127). When these survival signals are withdrawn or overwhelmed by strong stress signals (e.g., from engagement of Siglec-8, an inhibitory receptor), the cell may default to necroptosis if caspases are inhibited. Oxidative stress within the eosinophil itself, generated during the “respiratory burst, ” could also modify its own death machinery. A seminal study demonstrated that adhesion-induced eosinophil cytolysis requires the RIPK3-MLKL signaling pathway, while autophagy counteracts this process (126).

#### 3.3.2 Consequences: unleashing cytotoxic havoc

Eosinophil necroptosis leads to the uncontrolled release of cytotoxic contents, inflicting direct damage on airway structures and amplifying inflammation. Unlike the orderly packaging and disposal characteristic of apoptosis, necroptosis results in massive extracellular deposition of granule proteins, including eosinophil peroxidase (EPO), major basic protein (MBP), eosinophil cationic protein (ECP), and eosinophil-derived neurotoxin (EDN). MBP exhibits direct cytotoxicity toward airway epithelial cells by disrupting cell membranes (128), while ECP can form pores in target cells and possesses RNase activity that may perturb local RNA-sensing pathways. Beyond their inherent toxicity, these proteins function as endogenous alarmins, capable of directly activating epithelial cells and sensory nerves to induce bronchoconstriction, mucus hypersecretion, and further cytokine release (129), thereby creating a feedback loop that perpetuates eosinophil recruitment.

An additional consequential outcome is the formation of eosinophil extracellular traps (EETs). This process, termed ETosis, represents a distinct but potentially convergent mode of cell death. Certain stimuli, such as specific cytokines or complement components, may trigger a lytic death with features resembling necroptosis, involving reactive oxygen species and kinase activity (130). EETs consist of web-like structures of decondensed chromatin studded with cytotoxic granule proteins (131). Although physiologically intended to trap pathogens, in the pathological context of asthma, EETs become agents of tissue injury. They can directly damage the epithelium, present highly immunogenic stimuli, and form a persistent scaffold that sustains local inflammation and impedes tissue repair. Elevated levels of EETs have been clinically correlated with asthma severity and poor disease control (106, 107).

### 3.4 Airway smooth muscle cells and fibroblasts: architects of remodeling through death

Airway remodeling—encompassing airway smooth muscle (ASM) hypertrophy/hyperplasia, subepithelial fibrosis, and angiogenesis—is a cardinal feature of chronic asthma that correlates with fixed airflow obstruction and severity (132). Necroptosis of structural cells contributes to this process in paradoxical and profound ways.

#### 3.4.1 Airway smooth muscle cells: death begets growth

The notion of ASM cell death in a hyperplastic tissue seems contradictory. However, necroptosis in a subset of ASM cells can be a potent stimulatory signal. The lytic release of intercellular contents—including TGF-β1 (often in a latent complex that can be locally activated), fibroblast growth factor (FGF-2), and platelet-derived growth factor (PDGF)—creates a “death secretome” that stimulating proliferation, hyperplasia, and ECM production in neighboring surviving ASM cells and fibroblasts (18). It is important to note, however, that direct *in vivo* evidence for this mechanism specifically in asthmatic airway remodeling remains limited. Most supportive data come from *in vitro* co-culture experiments or from studies in other fibrotic diseases. In the asthmatic setting, ASM cells are exposed to chronic inflammatory cytokines (TNF-α, IL-1β) and oxidative stress, which can prime them for necroptosis (111, 112). The release of mitogenic and pro-fibrotic factors from necroptotic ASM cells is a compelling hypothesis that awaits definitive validation in animal models and human tissues. Nonetheless, the known capacity of TGF-β1 to drive ASM proliferation and ECM synthesis, together with the observation that RIPK3 expression is elevated in ASM bundles from severe asthmatics (113), supports the plausibility of this mechanism.

#### 3.4.2 Lung fibroblasts and myofibroblasts: depleting repair and seeding fibrosis

Fibroblasts are responsible for maintaining and repairing the ECM scaffold. Lung fibroblasts are sensitive to inflammatory cytokines and oxidative stress. Engagement of death receptors by ligands present in the asthmatic milieu (e.g., TNF-α, TRAIL) can induce necroptosis, especially under conditions of metabolic strain and ROS generation, as has been experimentally demonstrated in lung fibroblasts (133).

Necroptosis of fibroblasts depletes the repair cell pool. More insidiously, the contents of a necroptotic fibroblast are a rich source of pro-fibrotic signals. Beyond TGF-β, dying fibroblasts release connective tissue growth factor (CTGF/CCN2), a critical downstream mediator of TGF-β’s fibrotic effects, and IL-6, which promotes fibroblast proliferation. They also release lysyl oxidase (LOX) family members, particularly LOXL2, enzymes that cross-link collagen, making the fibrosis more stable and resistant to degradation (133, 134); LOXL2 has been identified as a key mediator upregulated in asthma and directly contributing to airway remodeling (135). Furthermore, the released cellular debris and DAMPs can activate nearby fibroblasts via TLRs, driving them into a persistently activated, pro-fibrotic state (136–138). This creates a feed-forward loop of fibrosis: inflammation causes fibroblast necroptosis, which releases factors that cause more fibroblast activation and matrix deposition, leading to progressive subepithelial fibrosis and airway stiffening.

## 4 Orchestrating the immune microenvironment: from cell death to disease endotype

The clinical heterogeneity of asthma—ranging from mild allergic disease to severe, steroid-refractory illness—has long puzzled clinicians and researchers (139). The emerging paradigm of asthma “endotypes” seeks to move beyond descriptive phenotypes to define distinct molecular pathways that drive disease. In this framework, necroptosis emerges not as a uniform background process, but as a master sculptor of the immune landscape. The specific cellular sources, triggers, and kinetics of necroptosis determine the precise cocktail of released mediators, which in turn instructs the recruitment, polarization, and sustained activation of immune cells. This chapter posits that divergent patterns of necroptotic activity constitute fundamental biological underpinnings for major asthma endotypes. We will dissect how a “DAMP-Alarmin Axis” driven by epithelial necroptosis fuels the type 2-high/eosinophilic endotype, and how a “DAMP-Inflammasome Axis” stemming from macrophage/immune cell necroptosis establishes the neutrophilic, steroid-resistant endotype. Finally, we will integrate how both axes converge, through chronic signaling and impaired resolution, to directly fuel the pathological structural changes of airway remodeling, thereby linking acute immunogenic cell death to long-term disease progression.

### 4.1 Driving the type 2-high, eosinophilic endotype: the epithelial-alarmin nexus

The classic allergic or early-onset eosinophilic asthma is characterized by high levels of IgE, IL-4, IL-5, IL-13, and prominent eosinophilic infiltration (140, 141). While adaptive Th2 cells are key players, the initiation and amplification of this response are increasingly attributed to innate immunity (142). Necroptosis of the bronchial epithelium provides a powerful, unified mechanism for this initiation.

#### 4.1.1 The initiating cascade: allergen-triggered epithelial necroptosis

The process begins with the inhalation of aeroallergens such as house dust mite (*Dermatophagoides pteronyssinus*), cockroach, or fungal spores (*Alternaria alternata*). As detailed in Chapter 2 and 3, these allergens possess intrinsic protease activity (e.g., Der p 1, cysteine proteases) that serve a dual function: breaching epithelial tight junctions and modulating cell death signaling. The functional consequence—reduced apoptotic capacity and a permissive state for necroptosis—has been observed in multiple experimental models (42, 43). While the precise molecular target(s) of allergen proteases on the death machinery remain incompletely defined, the net effect is a lowering of the necroptosis threshold. Concurrently, allergen proteases and particulate matter can activate epithelial G-protein-coupled receptors like protease-activated receptor-2 (PAR-2), leading to the production of endogenous “danger signals” such as reactive oxygen species (ROS) and the upregulation of TNF-α (143). In this primed state, with caspases functionally inhibited and death ligands present, the epithelium is exquisitely susceptible to RIPK1/RIPK3/MLKL-mediated necroptosis.

#### 4.1.2 The release of the “triad of alarmins” and innate immune activation

The lytic rupture of the epithelial cell in the massive, non-conventional release of three key epithelial-derived cytokines: Thymic Stromal Lymphopoietin (TSLP), IL-25, and IL-33. These are not merely released; they are released in their fully active or rapidly activatable forms (144). TSLP, released from necroptotic cells, acts on lung dendritic cells (DCs), programming them to migrate to draining lymph nodes and promote the differentiation of naïve T cells into a Th2 phenotype. It also directly activates mast cells and group 2 innate lymphoid cells (ILC2s) (145). IL-33, a nuclear sequestered in epithelial and endothelial cell nuclei, is a potent “alarmin.” Necroptosis causes its rapid release into the extracellular space, where it binds to the ST2 receptor on ILC2s, mast cells, basophils, and eosinophils, triggering potent activation. ILC2s activated by IL-33 (and IL-25) become prodigious producers of IL-5 and IL-13 (101, 146, 147). IL-25 also released upon epithelial damage, amplifies the type 2 response by promoting ILC2 expansion and function (148). The step from necroptosis to alarmin release is critical because it bypasses the need for initial adaptive immune recognition, providing an immediate innate signal that polarizes the immune response toward type 2 inflammation. This mechanism may explain both the rapid onset of symptoms upon allergen exposure in sensitized individuals and the presence of type 2 inflammation in very young children before their adaptive immune system is fully developed (130).

#### 4.1.3 Amplification and chronicity: the eosinophil feedback loop

Activated ILC2s and Th2 cells produce IL-5, which drives eosinophilopoiesis in the bone marrow and recruits’ eosinophils to the lung (149, 150). Eosinophils are not merely terminal effector cells but active participants within the inflammatory network. As detailed in Chapter 3.3, eosinophils recruited into this necroptosis- and alarmin-rich microenvironment become susceptible to necroptosis themselves, particularly as survival signals diminish or oxidative stress rises. Their lytic death releases cytotoxic granule proteins (MBP, ECP, EPO) and can lead to the formation of eosinophil extracellular traps (EETs) (151, 152). These cytotoxic proteins directly injure the airway epithelium, killing additional epithelial cells and initiating a feed-forward loop: more epithelial necroptosis generates more alarmins, which recruit more eosinophils; subsequent eosinophil necroptosis then exacerbates epithelial damage (17). Furthermore, cationic proteins such as MBP can activate sensory C−fibers in the airways, resulting in neurogenic inflammation, substance P release, and reflex bronchoconstriction—a mechanism that directly links cellular death to airway hyperresponsiveness (153).

The persistent release of intracellular proteins from necroptotic epithelial cells and eosinophils within a highly inflammatory milieu may also promote the presentation of self−peptides. For instance, cytokeratin fragments or eosinophil granule proteins could act as neoantigens, potentially driving a secondary autoimmune response that sustains inflammation even in the absence of allergen. This concept is supported by the detection of autoantibodies (e.g., against eosinophil peroxidase or nuclear antigens) in some patients with severe eosinophilic asthma. Thus, the type 2-high endotype can be conceptualized as a self-amplifying circuit propelled by necroptosis. This pathogenic axis is generally responsive to corticosteroids and biologics targeting IgE, IL-5, or IL-4Rα, as these agents act downstream of the initial necroptotic event (154).

### 4.2 Establishing the neutrophilic, steroid-refractory endotype: The DAMP-inflammasome axis

A significant subset of patients, often with late-onset, severe asthma, presents with airway inflammation dominated by neutrophils, a paucity of eosinophils, and a poor response to corticosteroids. This endotype is frequently associated with viral infections, air pollution, cigarette smoke exposure, and bacterial colonization (e.g., with *Haemophilus influenzae*) (155). Here, necroptosis plays a different, yet central, role, primarily through the activation of macrophages and a distinct downstream signaling cascade.

#### 4.2.1 Central role of macrophage and immune cell necroptosis

Under such pathological conditions, the primary necroptotic event frequently involves alveolar macrophages and may extend to airway epithelial cells, triggered by non−allergic stimuli. Viruses represent significant triggers; for instance, rhinovirus infection can induce macrophage necroptosis through the ZBP1/RIPK3 or TLR3/TRIF signaling pathways (see Section 2.2.4). Intracellular viral RNA sensors activate interferon responses, which upregulate components of the necroptotic pathway and prime cells for death (156–158). Bacterial components and environmental pollutants also contribute. Lipopolysaccharide (LPS) from colonizing bacteria sensitizes cells to TNF−α−induced necroptosis (159), while fine particulate matter (PM 2.5) drives necroptosis by inducing severe oxidative stress, inhibiting caspase activity, and promoting RIPK1/RIPK3 activation (160, 161). Necroptosis of macrophages releases a distinct profile of mediators compared to epithelial cells, including substantial amounts of pre-formed IL-1α, mature IL-1β (if the inflammasome is co-activated), TNF-α, and high-mobility group box 1 (HMGB1) protein in its disulfide (pro-inflammatory) form (162, 163).

#### 4.2.2 The NLRP3 inflammasome as the critical amplifier

This is where the immunopathological pathway diverges fundamentally from the type 2 axis. DAMPs released from necroptotic macrophages and epithelial cells potently activate the NLRP3 inflammasome in neighboring cells (164). For instance, extracellular ATP engages the P2X7 receptor, inducing potassium efflux, while oxidized mitochondrial DNA released from damaged mitochondria directly binds and activates NLRP3 (110). Additionally, crystalline structures derived from environmental pollutants or uric acid crystals released from dying cells provide a physical nucleation signal for inflammasome assembly. NLRP3 inflammasome activation leads to caspase−1−mediated processing of pro−IL−1β and pro−IL−18 into their active, secreted forms (105). IL-1β acts as the central cytokine of this axis. It drives epithelial cells and fibroblasts to produce potent neutrophil chemoattractants such as IL−8 (CXCL8) and CXCL1. Furthermore, within the inflammatory milieu rich in IL−6 and TGF−β, IL−1β promotes the differentiation of naïve T cells into Th17 cells (165).

#### 4.2.3 Establishment of the neutrophilic milieu and steroid resistance

The Th17/neutrophil axis plays a central role in this inflammatory cascade. Th17 cells secrete IL−17A and IL−17F, which further stimulate epithelial and mesenchymal cells to produce neutrophil chemoattractants and GM−CSF, thereby establishing a self−reinforcing loop that sustains neutrophil recruitment and survival (166, 167). Once recruited, neutrophils—which possess a short intrinsic lifespan—can themselves undergo necroptosis or NETosis (a distinct yet related form of lytic cell death) within this inflammatory milieu. This process releases neutrophil elastase and matrix metalloproteinase−9 (MMP−9), causing direct tissue injury, promoting mucus hypersecretion, and activating additional epithelial cells, which in turn perpetuates the inflammatory cycle (168–170).

A key feature of this endotype is its relative resistance to corticosteroids (CS), the mainstay of asthma therapy. Necroptosis offers a compelling mechanistic explanation for this clinical observation. First, CS primarily act on lymphocytes and eosinophils via genomic pathways but exhibit limited effects on neutrophil survival or macrophage function. More notably, corticosteroids may inadvertently promote necroptosis: they can upregulate the expression of TNF−α and other cytokines in macrophages and epithelial cells through the relief of NF-κB-mediated transcriptional repression (18). Additionally, by inhibiting gene transcription, CS may downregulate anti-apoptotic proteins such as cFLIP, which also suppresses necroptosis. This effectively lowers the threshold for RIPK1/RIPK3 activation, creating a paradoxical scenario in which treatment may exacerbate the very cell-death mechanism that drives inflammation (171). These mechanisms are context-dependent and may be most relevant in patients with underlying infection or high oxidative stress.

Thus, the neutrophilic asthma endotype is driven by a DAMP-inflammasome-Th17 axis. This axis demonstrates relative steroid resistance but may be amenable to therapeutic strategies targeting IL-1β (e.g., canakinumab) or specific necroptosis inhibitors (172–174).

### 4.3 Direct contribution to airway remodeling: the converging pathway of structural dysfunction

Beyond driving acute and chronic inflammation, the persistent necroptotic activity in both endotypes creates a microenvironment that directly and irreversibly alters airway structure—the process known as airway remodeling. This is the convergent point where the molecular pathways of necroptosis translate into fixed physiological impairment.

#### 4.3.1 The chronic “wound healing” milieu

The asthmatic airway, subjected to persistent necroptosis-driven inflammation, acquires features reminiscent of a chronic non−healing wound. A continuous release of growth factors from dying and activated cells generates a microenvironment rich in profibrotic and proliferative signals. Necroptotic epithelial cells, fibroblasts, and macrophages contribute to this milieu by releasing TGF−β1 (often in its latent form and subsequently activated by local proteases), platelet−derived growth factor (PDGF), and fibroblast growth factor (FGF−2) (136). Among these, TGF−β1 functions as a central regulator of fibrotic remodeling. Under the influence of TGF−β1 and the mechanical stress imposed by bronchoconstriction, resident lung fibroblasts differentiate into myofibroblasts that express α−smooth muscle actin (α−SMA). These activated cells exhibit a hyper−synthetic phenotype, excessively producing extracellular matrix (ECM) proteins such as collagen I, III, and V, fibronectin, and tenascin−C. This process underlies the characteristic subepithelial fibrosis and thickening of the reticular basement membrane observed in asthma (175). Concurrently, the airway smooth muscle (ASM) compartment undergoes structural changes. As outlined in Chapter 3.4, the secretome released from necroptotic ASM cells and other cellular sources delivers potent mitogenic signals—including PDGF and FGF—to neighboring surviving ASM cells. These signals drive ASM proliferation and hypertrophy, thereby expanding ASM mass. This increase directly contributes to airway−wall thickening and exacerbates bronchoconstrictor responses.

#### 4.3.2 Impaired resolution and autoimmunity

Necroptosis further contributes to the chronicity and persistence of asthma by impairing efferocytosis and promoting autoimmune-like responses. As noted previously, necroptosis of alveolar macrophages severely compromises efferocytic clearance (78). The resulting accumulation of apoptotic and necrotic cellular debris is not biologically inert; rather, it serves as a continual source of DAMPs that perpetuate inflammation. Furthermore, this debris can be phagocytosed by dendritic cells and presented as self-antigens, thereby linking innate immune events to adaptive immune activation. Within the highly oxidative and proteolytic environment of the asthmatic airway, self-proteins released during necroptosis can undergo post-translational modification. For instance, citrullination of structural proteins such as vimentin or actin fragments can generate novel antigenic epitopes (18). These neo−autoantigens may break immune tolerance and drive the production of autoantibodies. This mechanism is increasingly recognized in severe asthma, particularly in neutrophilic endotypes, and helps explain disease persistence even after removal of the initial external trigger (176). Ultimately, necroptosis acts as the initiating event that can shifts the pathology from a purely allergic toward an autoimmune−like phenotype.

#### 4.3.3 Angiogenesis and neural plasticity

Airway remodeling encompasses changes beyond fibrosis and smooth muscle enlargement. Within the inflammatory microenvironment, vascular endothelial growth factor (VEGF)—released from epithelial cells and macrophages—promotes angiogenesis, increasing the number and size of blood vessels in the airway wall. This vascular expansion contributes to edema and further thickens the airway wall (177). Additionally, mediators such as nerve growth factor (NGF) and brain-derived neurotrophic factor (BDNF), released from necroptotic cells and inflammatory, can alter the density and sensitivity of sensory nerves, thereby intensifying cough reflexes and bronchoconstriction (178–181).

### 4.4 Necroptosis−driven vicious cycles: beyond linear causality

The relationship between necroptosis and asthma pathogenesis is not linear but rather involves multiple bidirectional feedback loops. Recognizing these cycles is essential for understanding disease chronicity and for designing effective therapeutic interventions.

Cycle 1: The DAMP−Necroptosis Amplification Loop. Lytic release of DAMPs (ATP, HMGB1, mtDNA) from necroptotic cells activates neighboring cells via TLR4, P2X7, and other PRRs, inducing production of TNF−α, IL−1β, and type I interferons. These cytokines further upregulate necroptotic machinery (RIPK3, MLKL, ZBP1) and inhibit caspase−8, lowering the threshold for additional necroptosis (13). This creates a self−sustaining wave of cell death and inflammation.

Cycle 2: The Eosinophil−Epithelial Injury Loop. As described in Section 4.1.3, epithelial necroptosis recruits eosinophils; eosinophil necroptosis releases cytotoxic granules that injure the epithelium; injured epithelial cells undergo further necroptosis (182). This loop can persist even after the initial allergen trigger is removed.

Cycle 3: Impaired Efferocytosis−Necrosis Loop. Macrophage necroptosis reduces efferocytic capacity, leading to accumulation of apoptotic cells. These cells undergo secondary necrosis, releasing additional DAMPs that trigger more macrophage necroptosis (183). This loop is particularly relevant in severe, non−resolving asthma.

Cycle 4: Steroid Resistance−Necroptosis Loop. Corticosteroids, while effective in suppressing lymphocytic and eosinophilic inflammation, may under certain conditions (e.g., high TNF−α, infection) upregulate necroptotic pathway components and downregulate cFLIP, inadvertently promoting necroptosis (70). This necroptosis then drives steroid−resistant neutrophilic inflammation, creating a therapeutic challenge.

These vicious cycles highlight that necroptosis is not merely a consequence of inflammation but an active contributor that, once initiated, can perpetuate disease independently of the original trigger. Therapeutic interruption of any node within these cycles—whether by direct necroptosis inhibition, DAMP neutralization, or restoration of efferocytosis—holds promise for disease modification.

## 5 Therapeutic horizons and translational challenges: from mechanistic insight to clinical paradigm

The unequivocal delineation of necroptosis as a pathogenic cornerstone in asthma, particularly in severe and refractory forms, fundamentally shifts the therapeutic landscape. Moving beyond symptom control, targeting this lytic cell death pathway represents a paradigm-shifting strategy with genuine disease-modifying potential. This chapter critically evaluates the translational pipeline, from the preclinical and clinical development of specific pharmacological agents to the rational design of combination therapies. It confronts the imperative of patient stratification through biomarker discovery and honestly addresses the formidable scientific and clinical challenges that must be overcome to integrate “necroptosis inhibition” into the future asthma action plan.

### 5.1 Pharmacological inhibitors in development: precision targeting of the necroptotic cascade

The structured nature of the necroptosis pathway offers multiple nodes for pharmacological intervention. Drug development has progressed from tool compounds to clinical-grade candidates, each with distinct mechanistic implications and therapeutic profiles.

#### 5.1.1 RIPK1 kinase inhibitors: the first wave of clinical translation

RIPK1 functions as a critical signaling hub, integrating inputs from multiple death receptors and acting as a master switch governing cell survival, apoptosis, and necroptosis. Small-molecule inhibitors that selectively target the kinase activity of RIPK1 while preserving its essential scaffolding functions have emerged as leading therapeutic candidates. Compounds such as GSK2982772, R-552, and SAR443060 bind to the ATP-binding pocket of RIPK1, preventing its autophosphorylation and subsequent recruitment and activation of RIPK3, thereby aborting the formation of the necrosome. This upstream inhibition blocks not only lytic cell death but also the pro-inflammatory gene expression programs driven by RIPK1 kinase activity—a process termed “necroinflammation” (defined as the propagation of inflammation driven by the kinase activity of RIPK1 and the resulting transcriptional programs, independent of its role in cell death execution) (40).

Several RIPK1 inhibitors have advanced into Phase I and II clinical trials for autoimmune and inflammatory conditions including psoriasis, rheumatoid arthritis, and ulcerative colitis (184, 185). Their established human safety and pharmacokinetic profiles significantly accelerate their potential repurposing for asthma. A key consideration for pulmonary application is the route of administration. While oral formulations are under development, creating inhaled or nebulized versions is a primary objective. Local lung delivery would maximize target engagement within the airway epithelium and alveolar macrophages while minimizing systemic exposure, thereby reducing potential off-target effects and the risk of systemic immunosuppression (186). The principal advantage of RIPK1 inhibition lies in its broad blockade of pathway initiation; however, given RIPK1’s central role in multiple signaling networks, careful monitoring for potential disruptions to host defense and tissue homeostasis is warranted.

#### 5.1.2 RIPK3 and MLKL inhibitors: downstream specificity

Targeting components further downstream in the necroptotic cascade offers an alternative strategy with potentially greater specificity for the execution phase.

##### 5.1.2.1 RIPK3 inhibitors

Compounds like GSK872 and the repurposed anticancer drug dabrafenib directly prevent RIPK3 activation and its subsequent phosphorylation of MLKL (187). Preclinical evidence robustly supports their efficacy. In a murine model of house dust mite (HDM) -induced allergic asthma, administration of GSK872 significantly reduced levels of key type 2 cytokines (IL-4, IL-5, IL-13) in bronchoalveolar lavage fluid and serum, while attenuating airway inflammation, goblet cell hyperplasia, and subepithelial collagen deposition (18). These findings confirm that pharmacological RIPK3 inhibition can mitigate hallmark features of asthmatic pathology.

##### 5.1.2.2 MLKL inhibitors

Blocking the Final Common Pathway. Inhibiting the terminal effector MLKL represents arguably the most specific strategy, as its primary function is to execute plasma membrane rupture. Necrosulfonamide is a prototypical covalent inhibitor that binds to human MLKL at cysteine 86, preventing its membrane translocation and pore-forming activity (188). Next-generation, more drug-like MLKL inhibitors are under active investigation (189). The therapeutic appeal of this approach lies in preventing the catastrophic release of DAMPs and alarmins while potentially allowing upstream signaling events to proceed, thereby preserving some physiological functions of RIPK1 and RIPK3. Genetic ablation of MLKL in HDM-challenged mice results in a marked reduction airway inflammation and remodeling markers, providing genetic proof-of-concept for this strategy (190).

#### 5.1.3 Novel and repurposed agents targeting upstream triggers

Beyond the core necroptotic machinery, targeting upstream drivers presents complementary therapeutic opportunities. Disrupting the PTRF–IL-33–ZBP1 axis represents one such approach. Recent research has identified a pathway in which polymerase I and transcript release factor (PTRF/Cavin-1) in bronchial epithelial cells regulates IL-33 release. This IL-33, in turn, synergizes with HDM allergen to boost expression of Z-DNA binding protein 1 (ZBP1) and subsequent activation of the RIPK3/MLKL pathway in macrophages. This identifies PTRF and ZBP1 as novel, asthma-relevant targets (191). Inhibitors of this axis could specifically intervene in allergen-driven, ZBP1-mediated necroptosis, which operates partially independently of RIPK1 (78, 192–194).

Natural compounds with multi-target potential also hold promise. Bioinformatics-driven drug screening has identified natural products with affinities for key necroptosis-related proteins (195). Resveratrol and triptolide (a component of the traditional herb *Tripterygium wilfordii*) have been computationally predicted and experimentally shown to target proteins like NLRP3 and ALOX15, which are part of the necroptosis-associated network in asthma (15, 196–198). These compounds often possess additional anti-inflammatory and antioxidant properties, making them attractive multi-target candidates for further development.

### 5.2 Rational combination therapies: addressing pathological complexity

Given the intricate network of inflammatory and remodeling pathways in asthma, monotherapy targeting necroptosis may be insufficient, particularly in severe disease. Rational combination strategies are therefore designed to achieve synergistic efficacy by concurrently targeting parallel or sequential pathological processes.

#### 5.2.1 Combination with biologic agents: blocking initiation and amplification

This approach simultaneously suppresses the initial immunogenic cell death event and neutralizes the potent cytokines that perpetuate inflammation. Combining a RIPK1 inhibitor with an anti-alarmin biologic such as tezepelumab (anti-TSLP) or an anti-IL-33 antibody is biologically well-founded (199, 200). The necroptosis inhibitor reduces the lytic release of TSLP, IL-33, and IL-25 from the epithelium, while the biologic mops up any released alarmins, thereby establishing a dual barrier against the initiation of type 2 inflammation. In T2-high asthma, combining necroptosis inhibition with dupilumab (anti-IL-4Rα) could block both the upstream cell death that fuels inflammation and the downstream IL-4/IL-13 signaling responsible for IgE synthesis, eosinophilia, and mucus production. For neutrophilic asthma, combination therapy with anti-IL-1β or anti-IL-17 biologics may suppress the distinct inflammatory axis driven by necroptosis-mediated inflammasome activation (200).

#### 5.2.2 Combination with inflammasome inhibitors: quenching the inflammatory cascade

This combination is particularly rational for neutrophilic, steroid-resistant asthma. The potent and specific NLRP3 inflammasome inhibitor MCC950 blocks the production of IL-1β and IL-18 triggered by DAMPs (e.g., ATP and mtDNA) released from necroptotic cells (201–203). Administering it alongside a necroptosis inhibitor creates a powerful positive feedback loop breaker: reduced cell death leads to fewer DAMPs, resulting in diminished inflammasome activation, lower IL-1β production, and consequently attenuated neutrophil recruitment and activation.

#### 5.2.3 Combination with supportive agents: raising the threshold and managing symptoms

This strategy aims to enhance cellular resilience and address concurrent clinical symptoms. Given that oxidative stress is a key primer for necroptosis—mediated partly through caspase-8 inhibition—agents that ameliorate redox imbalance, such as N-acetylcysteine or NRF2 activators, could raise the threshold for necroptosis induction and potentially enhance the efficacy of a direct inhibitor (204, 205). Furthermore, since excessive mucus production is a major contributor to morbidity, combining a necroptosis inhibitor with a mucus modulator like niclosamide, which has demonstrated efficacy in inhibiting hypersecretion, targets both the inflammatory driver and a debilitating symptom (206–208). The rational combination strategies are summarized in **Table 1**.

**Table 1:** Rational combination therapy strategies for asthma endotypes.

Combination strategy	Example agents	Targeted asthma endotype	Mechanistic rationale	References
Necroptosis Inhibitor + Biologic	RIPK1i (GSK2982772) + Anti-TSLP (Tezepelumab)	T2-High, Allergic	Blocks immunogenic death & neutralizes key released alarmin.	(184–186)
Necroptosis Inhibitor + Inflammasome Inhibitor	MLKLi+NLRP3i (MCC950)	Neutrophilic, Steroid-Resistant	Blocks DAMP release & inhibits consequent IL-1β/IL-18 production.	(185, 201, 203)
Necroptosis Modulator + Supportive Agent	RIPK1i+High-dose N-acetylcysteine	Mixed/Severe	Inhibits death pathway & reduces oxidative stress that primes cells for death.	(185, 204)

### 5.3 Biomarkers for patient stratification: the quest for the “necroptosis-high” endotype

The successful clinical translation of therapies targeting necroptosis hinges entirely on the ability to identify the patient subgroup whose disease is predominantly driven by this pathway—the so−called “necroptosis−high” endotype. Achieving this requires a multimodal biomarker strategy.

#### 5.3.1 Direct pathway biomarkers

A promising approach involves measuring phosphorylated forms of the key necroptotic effectors, p−RIPK3 and p−MLKL, which can be detected within circulating extracellular vesicles (e.g., exosomes) released from dying lung cells. Their levels in serum or plasma could provide a quantifiable, real−time readout of ongoing pulmonary necroptotic activity (209, 210). Supportive, albeit indirect, biomarkers may include elevated systemic levels of DAMPs such as HMGB1, S100 proteins, or cell−free mitochondrial DNA. Sampling from the lung compartment itself, such as sputum supernatant or exhaled breath condensate, could reveal local indicators. For instance, shifts in the cystine/cysteine redox couple or the presence of specific lipid peroxidation products may signal the reductive and oxidative stress that predisposes cells to necroptosis. Proteomic analysis of bronchoalveolar lavage fluid could further identify unique protein fragments generated by necroptosis−specific cleavage.

#### 5.3.2 Transcriptomic and genetic signatures

Machine−learning analysis of airway−tissue transcriptomics is helping to define necroptosis−related gene signatures. A 2024 study, for example, identified a core set of four necroptosis−related differentially expressed genes in asthma: upregulated *NLRP3, PYCARD, and ALOX15*, alongside downregulated VDAC3 (15). Notably, the expression levels of these genes correlated with key clinical parameters: NLRP3 expression was associated with worse lung function (FEV1/FVC), while ALOX15 expression correlated with elevated fractional exhaled nitric oxide, sputum and blood eosinophil counts, and IgE levels. A composite “Necroptosis Score” derived from such signature genes—measurable in bronchial biopsies or even in sorted sputum cells—could serve as a powerful tool for patient stratification.

### 5.3 Functional cellular assays

Functional assessment of a patient’s cellular propensity for necroptosis represents another stratification strategy. This can be achieved by challenging patient−derived cells—such as peripheral blood mononuclear cells or airway macrophages—ex vivo with established necroptosis inducers (e.g., TNF−α combined with the pan−caspase inhibitor zVAD−fmk). The degree of cell death under these conditions would act as a functional cellular biomarker, reflecting an inherent susceptibility to this pathway (20).

### 5.4 Challenges and future directions: bridging the translational gap

Despite the compelling mechanistic rationale, translating necroptosis−targeted therapies into clinical practice for asthma faces significant translational hurdles that must be systematically addressed through focused research.

#### 5.4.1 Defining the therapeutic window

A pivotal clinical question centers on the optimal timing for therapeutic intervention. It remains unclear whether necroptosis inhibitors should be deployed as a chronic maintenance therapy in biomarker−positive severe patients to prevent subclinical epithelial injury and remodeling, as an exacerbation−preventive strategy initiated at the first sign of an upper respiratory infection to block virus−triggered epithelial necroptosis, or as an acute treatment during severe inflammatory crises. Preclinical models that accurately mimic these distinct clinical scenarios are urgently needed to guide therapeutic strategy.

#### 5.4.2 Safety and host defense concerns

Given the evolutionary role of necroptosis in defending against intracellular pathogens, legitimate concerns exist regarding the potential for increased susceptibility to viral and bacterial infections with long-term systemic inhibition of RIPK1 or RIPK3. For example, in a murine model of severe influenza, necroptosis blockade prevented lethal lung injury but also delayed viral clearance, highlighting a delicate balance between controlling inflammation and maintaining host defense (211). This underscores the critical importance of developing inhaled, lung-targeted formulations to limit systemic immunosuppression. Furthermore, comparative safety studies are required to understand the infection-related risks of inhibiting upstream regulators like RIPK1 versus the terminal executor MLKL (212). It is possible that MLKL inhibition carries a lower risk of immunosuppression, as it does not interfere with the scaffolding or NF-κB functions of RIPK1.

#### 5.4.3 The biomarker imperative

The risk of clinical trial failure due to enrollment of heterogeneous patient populations necessitates a concerted effort to prospectively validate candidate biomarkers. Promising candidates, such as p−MLKL−positive extracellular vesicles or the necroptosis−related gene expression signature, require rigorous validation in large, well−phenotyped severe asthma cohorts. The ultimate goal is to develop a clinically feasible diagnostic test capable of reliably identifying the “necroptosis−high” endotype. Without such a test, even the most potent necroptosis inhibitor may fail in an unselected asthma population.

#### 5.4.4 Bridging the translational gap: the need for human evidence

As noted throughout this review, the majority of mechanistic insights derive from *in vitro* cell culture and murine models. Species differences exist—for example, human MLKL contains a cysteine residue (Cys86) that is absent in mouse MLKL, affecting inhibitor sensitivity (160). Moreover, the inflammatory microenvironment of the human asthmatic lung, with its complex mixture of cytokines, proteases, and oxidative species, may not be fully recapitulated in mouse models. To address these limitations, future research must prioritize several human tissue-based approaches. First, single-cell and spatial transcriptomics on human bronchial biopsies can precisely map the expression of necroptotic machinery to specific cell types in disease. Second, ex vivo precision-cut lung slices from asthmatic patients undergoing lung resection allow direct assessment of necroptosis inhibitor efficacy in a human tissue context. Third, longitudinal cohorts with serial sampling of blood and sputum are needed to correlate candidate biomarkers with disease activity and treatment response.

#### 5.4.5 Future research trajectories

Several key research directions will be essential to bridge the translational gap. First, single−cell and spatial transcriptomics on human bronchial biopsies will precisely map the expression of necroptotic machinery to specific cell types, thereby identifying the key cellular drivers in human disease. Second, advanced preclinical models incorporating sequential viral and allergic insults, along with cell−type−specific genetic manipulations, are needed to establish causal relationships and model exacerbations. Third, research should investigate whether persistent necroptosis contributes to autoantibody generation in severe asthma, potentially through chronic antigen release and impaired efferocytosis, which could open novel therapeutic avenues. Finally, given the interconnected nature of cell death pathways (Section 2.3), future studies should explore whether co−targeting necroptosis and pyroptosis (e.g., a RIPK3 inhibitor plus a gasdermin D inhibitor) yields synergistic benefits compared to targeting either pathway alone.

In summary, while the necroptosis paradigm offers a transformative framework for understanding and treating asthma, its clinical translation depends on parallel progress in developing safe, targeted inhibitors, validating precise biomarkers for patient selection, and designing clinical trials for the appropriate patient at the optimal time.

## 6 Conclusion and future perspectives

### 6.1 The necroptosis nexus: a unifying framework for asthma heterogeneity

The clinical and molecular heterogeneity of asthma has long posed a challenge for diagnosis and therapy. The necroptosis framework provides a powerful, unifying logic that explains how diverse environmental triggers converge on a common pathological pathway yet yield distinct disease endotypes based on cellular context and the resulting immunogenic output. This paradigm elegantly connects the initial insult—whether an allergen−derived protease, viral RNA, or particulate matter—to the dismantling of cellular safeguards and the activation of the RIPK1−RIPK3−MLKL axis. The subsequent divergence into major endotypes is dictated by the cellular source of death: epithelial necroptosis drives the type 2−high phenotype via alarmin release, while macrophage/immune cell necroptosis propels the neutrophilic, steroid−resistant phenotype through DAMP−mediated inflammasome activation. Chronically, both pathways feed into airway remodeling through the persistent release of growth factors and the impairment of efferocytosis.

Importantly, this framework does not position necroptosis as an isolated actor. As discussed in Section 2.3, the asthmatic airway is a site of complex interplay among multiple RCD pathways—pyroptosis, ferroptosis, and autophagy—often operating within the PANoptosis paradigm. Necroptosis frequently serves as the initial trigger that engages these other programs, creating a web of feed-forward amplification. The bidirectional vicious cycles outlined in Section 4.4 (DAMP-necroptosis loop, eosinophil-epithelial loop, impaired efferocytosis loop, and steroid resistance loop) further illustrate that necroptosis is not merely a downstream consequence of inflammation but an active participant that can sustain disease independently of the original trigger. Thus, necroptosis acts as the common pathological soil from which the varied phenotypes of asthma grow, offering a cohesive narrative from environmental exposure to immune polarization and structural damage.

### 6.2 Therapeutic implications: from symptom control to disease modification

Targeting necroptosis represents a paradigm shift from current strategies that largely inhibit downstream cytokines, moving intervention upstream to the root of immunogenic cell death. This approach holds the promise of true disease modification. The well−defined molecular cascade offers multiple druggable nodes, including upstream RIPK1 kinase inhibitors (e.g., GSK2982772, R-552) and execution−specific MLKL blockers (e.g., necrosulfonamide derivatives), with preclinical studies demonstrating efficacy in reducing inflammation, goblet cell hyperplasia, and subepithelial collagen deposition in murine asthma models (189). The development of inhaled or nebulized formulations is paramount to maximize lung-targeted efficacy while mitigating systemic immunosuppression risks, making local delivery a priority for long-term therapeutic use (209). However, the path to clinical translation is not without obstacles. As noted in Section 5.4, the evolutionary role of necroptosis in host defense raises legitimate concerns about increased susceptibility to viral and bacterial infections with chronic systemic inhibition. The optimal therapeutic window—chronic maintenance, exacerbation prevention, or acute treatment—remains undefined and likely varies by endotype. These challenges underscore the need for careful patient selection and, ideally, lung-targeted drug delivery.

### 6.3 The translational roadmap: key challenges and future directions

Bridging the gap from mechanistic insight to clinical application requires a focused agenda, with biomarker development as the highest priority. The success of targeted therapy depends entirely on identifying the “necroptosis−high” endotype through liquid biopsies that detect phosphorylated RIPK3 or MLKL in circulating extracellular vesicles (213), airway−derived transcriptomic signatures such as the four−gene panel (NLRP3, PYCARD, ALOX15, VDAC3) that correlates with lung function and eosinophil counts, and functional cellular assays measuring the necroptotic propensity of patient−derived macrophages or bronchial epithelial cells upon ex vivo challenge. Human tissue validation is critically needed, as most current evidence derives from murine models and cell lines; species differences in MLKL regulation and necroptotic signaling between human and mouse necessitate verification in human samples. Accordingly, future studies should employ single−cell and spatial transcriptomics on human bronchial biopsies, ex vivo precision−cut lung slices from asthmatic patients, and longitudinal biomarker−focused cohorts.

Further priorities include exploring the PANoptosis connection, as the boundaries between necroptosis, pyroptosis, and apoptosis in the asthmatic airway are likely fluid (210). Research should determine whether co−targeting multiple RCD pathways (e.g., RIPK3 plus gasdermin D inhibitors) yields synergistic benefits or merely shunts cells toward alternative death programs. Finally, autoimmunity as a consequence of chronic necroptosis remains an understudied hypothesis: the persistent release of modified self−proteins (e.g., citrullinated vimentin) from necroptotic cells, combined with impaired efferocytosis, could break immune tolerance. Whether this contributes to autoantibody responses in severe asthma warrants direct investigation.

### 6.4 Final synthesis: toward a new therapeutic era

The recognition of necroptosis as a central pathogenic engine in asthma provides a transformative and unifying framework for the disease’s heterogeneity and chronicity. While translational challenges in biomarker development, drug delivery, and trial design remain substantial, the trajectory points toward a future of precision medicine. By moving upstream to silence the initiating signal of immunogenic cell death, this approach offers the potential to fundamentally alter the disease course, prevent irreversible remodeling, and achieve transformative outcomes for patients with severe and refractory asthma, thereby illuminating a clear path toward a new era of disease−modifying therapy.
